# Prognostic impact of imported and newly-isolated methicillin-resistant *Staphylococcus aureus *in the ICU

**DOI:** 10.1186/cc9653

**Published:** 2011-03-11

**Authors:** S Ohshimo, K Ota, T Tamura, Y Kida, J Itai, K Suzuki, T Inagawa, Y Torikoshi, T Otani, T Sadamori, R Tsumura, Y Iwasaki, N Hirohashi, K Tanigawa

**Affiliations:** 1 Hiroshima University, Hiroshima, Japan

## Introduction

Methicillin-resistant *Staphylococcus aureus *(MRSA) is a leading pathogen of hospital-acquired pneumonia. The difference in outcome between patients with imported and newly-isolated MRSA in the ICU has not been well investigated. The aim of our study was to explore the incidence, risk factors and outcome in patients with imported and newly-isolated MRSA.

## Methods

Patients admitted to the ICU in our university between April 2009 and May 2010 were prospectively studied. Nasal swabs were collected from all patients on admission and subsequently collected weekly during the ICU stay. When patients were intubated, intratracheal aspirates were concurrently collected. The correlations of positive culture of MRSA with clinical variables were analyzed.

## Results

A total of 1,270 consecutive patients were enrolled. The median follow-up period was 404 days (range, 187 to 609). There were 803 males and 467 females. Median age was 63 (range, 1 to 97). Of these, imported MRSA was found in 124 (10%) patients, and newly-isolated MRSA in 57 (4%) patients. The incidence of imported MRSA was associated with the co-morbidity of cardiovascular disease or malignancy and long hospital stay before admission to the ICU, whereas the incidence of newly-isolated MRSA was associated with the positive culture in intratracheal aspirates or blood/intravenous catheter, the co-morbidity of shock, pneumonia, neurological diseases or trauma, increased number of isolated sites, higher APACHE II score, prolonged ICU stay and higher mortality during ICU stay. Although no statistical significance was found in total patients, the subset analysis of the male patients demonstrated that the outcome of newly-isolated patients was significantly poor compared with those of imported MRSA (*P *= 0.005). Multivariate analysis revealed that the new isolation of MRSA in the ICU (*P *= 0.03; hazard ratio (HR), 2.62), negative culture of MRSA in nasal swab (*P *= 0.02; HR, 4.18), ≥2 isolated sites (*P *= 0.01; HR, 4.59) and co-morbidity of ARDS (*P *= 0.002; HR, 4.63) were the independent poor prognostic factors.

## Conclusions

New isolation of MRSA during the ICU stay was associated with poor outcome particularly in male patients compared with imported MRSA. Clinicians should be aware of the high-risk group of MRSA infection. Strict hand hygiene plus a careful assessment of the patient, applying aggressive procedures such as patient isolation, staff cohorting, and active surveillance cultures should be indicated.

